# Genomic Landscape in Neoplasm-Like Stroma Reveals Distinct Prognostic Subtypes of Pancreatic Ductal Adenocarcinoma

**DOI:** 10.3389/fonc.2021.771247

**Published:** 2021-10-18

**Authors:** Jiahong Jiang, Yaping Xu, Lianpeng Chang, Guoqing Ru, Xuefeng Xia, Ling Yang, Xin Yi, Zheling Chen, Dong-Sheng Huang, Liu Yang

**Affiliations:** ^1^ Key Laboratory of Tumor Molecular Diagnosis and Individualized Medicine of Zhejiang Province, Zhejiang Provincial People’s Hospital, People’s Hospital of Hangzhou Medical College, Hangzhou, China; ^2^ Department of Oncology, Zhejiang Provincial People’s Hospital, People’s Hospital of Hangzhou Medical College, Hangzhou, China; ^3^ Geneplus-Beijing Institute, Beijing, China; ^4^ Department of Pathology, Zhejiang Provincial People’s Hospital, People’s Hospital of Hangzhou Medical College, Hangzhou, China

**Keywords:** pancreatic cancer, tumor microenvironment, stroma, *KRAS*, disease-free survival

## Abstract

As a main component of the tumor microenvironment, the stroma is critical in development, progression, and metastasis of pancreatic ductal adenocarcinoma (PDAC). The genomic status and its relationship of neoplastic and stromal components remain unclear in PDAC. We performed targeted sequencing for 1,021 cancer-suspected genes on parallel microdissected stromal and neoplastic components from 50 operable PDAC patients. Clonality analysis of mutations was conducted to reconstruct the evolutionary trajectory, and then molecular subtypes were established. Multi-lineage differentiation potential and mesenchymal transformation of *KRAS*-mutant cell line Panc1 were evaluated using RT-PCR and immunofluorescence staining. In this study, 39 (78.0%) were genomically altered in stroma, with *KRAS* (71.8%), *TP53* (61.5%), and *CDKN2A* (23.1%) as the most commonly mutated genes. The majority of stromal mutations (89.8%) were detected in matched neoplastic components. Patients with *KRAS/TP53*-mut stroma demonstrated a higher tumor cell fraction (TCF) than did those with wild-type (WT) stroma (*p* = 0.0371, *p* = 0.0014). In both components, mutants *KRAS* and *TP53* often occurred as clonal events, and the allele frequencies presented linear correlation in the same specimen. All neoplasm-like stroma (characterized with all or initial neoplastic clones and driver events in stroma) harbored *KRAS* or *TP53* mutations. Neoplasm-like and *KRAS*-mutant stroma was associated with shorter disease-free survival. It is a new finding for the existence of driver gene mutations in PDAC stroma. These data suggest that genomic features of stromal components may serve as prognostic biomarkers in resectable PDAC and might help to guide a more precise treatment paradigm in therapeutic options.

## Background

Despite intense efforts over the last decade, pancreatic ductal adenocarcinoma (PDAC) is still considered one of the most aggressive and lethal solid tumors (1). Most patients who present with advanced PDAC will die within a year of diagnosis. Even with resectable PDAC, patients have a 5-year overall survival of only 15% to 25% after radical resection and adjuvant chemotherapy (2). Lack of effective markers for prognosis prediction and precision treatment is also attributed to the high morality. As the only monitoring marker permitted by the Food and Drug Administration (FDA), carbohydrate antigen 19-9 (CA19-9) is easily affected by biliary disease and is negative in Lewis (−) PDAC patients (3). Recent studies have proposed some biomarkers to predict prognosis of PDAC; however, none of them achieved satisfying results (4). In this condition, new biomarkers are urgently needed to guide precise treatment and predict prognosis for PDAC.

The tumor microenvironment (TME) is a complicated network that contains blood and lymphatic vessels, immune cells, stromal cells, and extracellular matrix (cytokines, growth factors, chemokines, and inflammatory factors). The dynamic communication between cancer cells and TME influences cancer proliferation, invasion, metastasis, drug resistance, and immune escape. Immune cells such as tumor-associated macrophages have critical functions in tumor development through manifold growth factor secretion and numerous immunosuppressive molecule production (5). As a critical component of the TME, the tumor stroma has a profound effect on many hallmarks of cancer (6). High stromal component in PDAC was confirmed as an independent prognostic factor through digitalized whole-mount histopathology, as well as the impact of tumor grade and perineural invasion (7). Therefore, exploring the characteristics of the stroma will help in addressing the progression and metastasis of PDAC.

Epithelial–mesenchymal transition (EMT) has been proposed as an important interactive way between tumor and stroma during malignant progression (8). Recent studies have reported that a mesenchymal or epithelial phenotype is not a stable property of cancer cells and is often defined by the gain of the mesenchymal marker vimentin and the loss of the epithelial marker E-cadherin. EMT is a process in which epithelial cells acquire mesenchymal features, with enhanced capacity of invasion and metastasis in cancer. Epithelially derived cells were observed to migrate into the stroma and transformed to mesenchymal phenotype using lineage tracing mouse model at early stage of PDAC (9). Based on this study, we guessed that epithelially derived cells might affect the genomic features of stroma *via* EMT. Whether genomic features of the stroma have prognostic value for PDAC is an issue of concern.

To identify genomic mutations in stroma components and to evaluate their prognostic value in patients with resectable PDAC, this prospective study collected surgical tissue samples from 50 patients with PDAC. We used laser capture microdissection (LCM) technique to separate stroma from neoplastic components, and then we performed next-generation sequencing (NGS) for both specimens. Clinical characteristics and clonality analysis of mutations were conducted to explore the role of stroma in PDAC, and *in vitro* experiments were performed to clarify this condition.

## Materials and Methods

### Clinical Cohort

This single-center prospective study was conducted at Zhejiang Provincial People’s Hospital. From May 2016 to November 2016, a total of 50 patients primarily diagnosed with PDAC and received surgery were enrolled in this study. The database was locked for follow-up and analyses on November 2018. Patients with a concurrent malignant neoplasm were excluded. The histopathological status was evaluated by at least two experienced pathologists. TNM staging was defined according to American Joint Committee on Cancer (AJCC) TNM staging system for pancreatic cancer (10). Radiographic assessment using the Response Evaluation Criteria in Solid Tumors (RECIST) version 1.1 was performed and based on standard of care clinical guidelines. We followed the Strengthening the Reporting of Observational Studies in Epidemiology reporting guideline statement to ensure the quality of data reported in this study (11). This study was approved by the ethical committee at Zhejiang Provincial People’s Hospital (No. 2016KY129). All patients provided informed written consent before undergoing any study-related procedures. This study was performed in accordance with the Declaration of Helsinki.

### Sample Collection

Surgical tumor tissue samples and blood lymphocytes were collected from each patient. Tissue samples were fixed by formalin-fixed paraffin-embedded (FFPE). H&E staining was performed for each section according to the manufacturer’s instructions (Beyotime, Suzhou, China) before dissection. As a guide for stromal lesion, we performed immunohistochemistry staining for vimentin (Cat #5741 purchased from Cell Signaling Technology, Danvers, MA, USA) in the adjacent section. Ten to 15 sections of FFPE (thickness: 5–10 μm) were cut using a microtome (RM2265, Leica, Germany). LCM was performed to separate the neoplastic components, fibrotic stroma, and normal pancreatic tissue on a Leica LMD7000 microscope as previously described (12). The microdissection was performed by at least two senior pathologists, and any disagreement between these pathologists was resolved by discussion. All specimens were then stored in phosphate-buffered saline (PBS) before subsequent processing.

### DNA Extraction

Genomic DNA was extracted from neoplastic, stromal, and normal components using a QIAamp DNA Mini Kit (Qiagen, Hilden, Germany). Germline DNA was extracted from blood lymphocytes using the DNeasy Blood & Tissue Kit (Qiagen, Hilden, Germany). DNA quality was estimated using a Qubit fluorometer and a Qubit dsDNA (BR) Assay Kit (Invitrogen, Carlsbad, CA, USA). Genomic DNA from normal tissues and germline DNA from blood lymphocytes were used as negative control to eliminate the interference of germline variants and contaminating cancer cells.

### Sequencing Library Constructing

Extracted DNA was sheared into 200- to 250-bp fragments *via* a Covaris S2 instrument (Woburn, MA, USA). KAPA LTP Library Preparation Kit for Illumina (KAPA Biosystems, Boston, MA, USA) was used to prepare indexed NGS libraries. NEBNext FFPE DNA Repair Mix (Ipswich, UK) was used for FFPE DNA repair during library construction, and the detailed protocol can be obtained from https://international.neb.com/protocols/2015/01/16/protocol-for-use-with-nebnext-ffpe-dna-repair-mix-m6630-and-other-user-supplied-library-construction-reagents. Additional information regarding library preparation was described by Lv et al. (13).

### Targeted Capture Sequencing

Libraries were hybridized to custom-designed biotinylated oligonucleotide probes (Integrated DNA Technologies, IA, USA). The captured genomic regions included the most common driver genes of solid tumors (14). We chose their entire exome regions to construct the basic panel. Next, genomic regions related relevant to the effects of chemotherapy, targeted drugs, and immunotherapy per available clinical and preclinical research were added to the panel. Finally, high-frequently mutant regions recorded in the Catalogue of Somatic Mutations in Cancer (COSMIC, http://cancer.sanger.ac.uk/cosmic) and The Cancer Genome Atlas (TCGA; https://cancergenome.nih.gov/) were involved. Overall, 1,021 genes were involved in this panel. Sequencing was carried out using Illumina 2 × 100-bp paired-end reads on an Illumina HiSeq 3000 instrument according to the manufacturer’s recommendations using a TruSeq PE Cluster Generation Kit v3 and a TruSeq SBS Kit v3 (Illumina, San Diego, CA, USA). Additional detailed information regarding library preparation was described by Lv et al. (13). The median sequencing depth of stromal and neoplastic components was 941× (360× to 1,626×) and 1,045× (345× to 1728×), respectively.

### Raw Data Processing

Terminal adaptor sequences and reads with more than 50% low-quality base reads, or those with more than 50% N bases, together with their mate pair were removed from raw reads. Subsequently, Burrows–Wheeler Aligner (BWA; version 0.7.12-r1039, http://bio-bwa.sourceforge.net/) tool was used to align clean reads to the reference human genome (hg19) with default parameters. Duplicate reads were identified and marked with Picard’s Mark Duplicates tool (https://software.broadinstitute.org/gatk/documentation/tooldocs/4.0.3.0/picard_sam_markduplicates_MarkDuplicates.php). The Gene Analysis Toolkit (GATK, https://www.broadinstitute.org/gatk/) was used to perform local realignment and base quality recalibration.

### Somatic Mutation Calling

Somatic single-nucleotide variations (SNVs) and insertions or deletions of small fragments (indels) were called using the MuTect2 algorithm (https://software.broadinstitute.org/gatk/documentation/tooldocs/3.8-0/org_broadinstitute_gatk_tools_walkers_cancer_m2_MuTect2.php). The filter criteria included 1) variants supported by fewer than five high-quality reads (base quality ≥30, mapping quality ≥30) were filtered; 2) variants were filtered as cross-contamination if present in >1% samples in custom single-nucleotide polymorphism (SNP) databases (dbsnp, https://www.ncbi.nlm.nih.gov/projects/SNP/; 1000G, https://www.1000genomes.org/; ESP6500, https://evs.gs.washington.edu/; ExAC, http://exac.broadinstitute.org/) and self-built SNP database; 3) synonymous mutations (also listed in **Table S1**) were removed; 4) variants with allele frequency less than 1% were removed; and 5) variants detected in matched blood lymphocytes and normal tissue were removed. The final candidate variants were all manually verified in the Integrative Genomics Viewer (IGV; https://igv.org/). Remaining mutations were considered validated somatic variants.

### Determination of Driver Mutations

Two steps were performed to determine driver or passenger mutations. First, evidential driver genes of PDAC were determined according to Bailey et al. (15). Second, Polymorphism Phenotyping v2 (PolyPhen-2, http://genetics.bwh.harvard.edu/pph2/) and Sortig Intolerant From Tolerant (SIFT, http://sift.bii.a-star.edu.sg/) were used to predict whether the protein structural change derived by one mutation was harmful or not. Those with PolyPhen-2 Score >0.85 or SIFT Score <0.05 were defined as harmful mutations. Generally, harmful mutations in driver genes were defined as driver events, and the others, including harmless mutations in driver genes and all mutations in passenger genes, were defined as passenger events.

### Clonality Analysis

PyClone algorithm was used to determine the clonal clusters (16). The essential parameters included the variant allele frequencies (VAFs) and copy numbers of non-synonymous mutations in both tumor and stromal components. Copy number was estimated by Contra algorithm (http://contra-cnv.sourceforge.net).

### Cell Culture and Reagents

Human pancreatic cancer cell line Panc1 was purchased from American Type Culture Collection (Manassas, VA, USA), and it was grown in DMEM culture medium (Gibco, Carlsbad, CA, USA) supplemented with 10% fetal bovine serum (FBS) and 1% penicillin/streptomycin, in a humidified atmosphere at 37°C and 5% CO_2_.

### Reverse Transcriptase PCR

Total RNA was extracted from cells using TRIzol reagent (Invitrogen, California, USA) according to the manufacturer’s instruction. cDNA was subsequently synthesized using PrimeScript™ RT Master Mix kit (Takara). RT-PCR was performed using SYBR Premix Ex Taq™ kit (Takara, Dalian, China). The primers are listed in **Table S2**.

### Immunofluorescence Staining

Panc1 cells were seeded on coverslips and cultured under different glycemic conditions for 3 days. Then medium was removed, and vimentin was stained using the rabbit anti-human vimentin antibody (Cat #5741) purchased from Cell Signaling Technology (Danvers, MA, USA). Evaluation was performed using confocal laser scanning microscope (Leica, Wetzlar, Germany).

### Statistical Analysis

Descriptive statistics was performed using SPSS 22.0 (IBM, Armonk, NY, USA). Spearman’s correlation analysis was performed using GraphPad Prism 7 (GraphPad Software, La Jolla, CA, USA) to assess the relevance of VAFs between mutants *KRAS* and *TP53*, as well as the correlation between tumor cell fraction (TCF) and maximal VAF in stromal or neoplastic component. The parameter comparison for different patient groups was performed using the Mann–Whitney U-test (two groups) or one-way ANOVA test (≥3 groups) (SPSS 22.0). The Kaplan–Meier survival analysis was used to compare disease-free survival (DFS) between different subgroups, and Cox regression was performed to determine the influence of multiple factors on DFS. Both analyses were conducted by SPSS 22.0. A two-tailed *p-*value <0.05 was considered statistically significant.

## Results

### Clinical Characteristics

Clinical characteristics of patients are summarized in **Table 1**. All of 50 patients were diagnosed with primary PDAC. The median age at diagnosis was 65 years (ranged from 37 to 84 years). The number of male and female patients was 30 (60.0%) and 20 (40.0%), respectively. The majority of enrolled patients were stage I/II (n = 44, 88.0%). Histologically, 25 patient specimens (50.0%) were poorly differentiated in terms of cellular morphology, and the others were moderately differentiated. The maximal diameter of tumor *in situ* was >4 cm in seven patients (14.0%). Regional lymph nodes were involved in 19 patients (38.0%). The adjacent nerve and vasculature were invaded in 41 (82.0%) and 21 (42.0%) of patients, respectively.

**Table 1:** Correlation between clinical characteristics and genomic status of stroma.

Characteristics	Any mutation (n = 50)			Mutant *KRAS* (n = 50)			Mutant *TP53* (n = 50)			Co-mutants *KRAS* and *TP53* (n = 50)			
	Positive (n = 39)	Negative (n = 11)	*p*	Positive (n = 28)	Negative (n = 22)	*p*	Positive (n = 24)	Negative (n = 26)	*p*		Positive (n = 21)	Negative (n = 29)	*p*
**Age, years**													
Median	66	65	0.3325	68	62.5	0.0036*	68.5	63.5	0.0101*		69	63	0.0029*
**Gender, n (%)**													
Male	23 (59)	7 (64)	0.9444	15 (54)	15 (68)	0.2952	13 (54)	17 (65)	0.4186		10 (48)	20 (69)	0.1283
Female	16 (41)	4 (36)		13 (46)	7 (32)		11 (46)	9 (35)			11 (52)	9 (31)	
**Differentiation, n (%)**													
Poor	19 (49)	6 (55)	0.7328	14 (50)	11 (50)	1.0000	13 (54)	12 (46)	0.5713		11 (52)	14 (48)	0.7745
Other	20 (51)	5 (45)		14 (50)	11 (50)		11 (46)	14 (54)			10 (48)	15 (52)
**Clinical stage, n (%)**													
I	22 (56)	3 (27)	0.0878	15 (54)	10 (45)	0.5688	15 (63)	10 (38)	0.0894		12 (57)	13 (45)	0.3900
II-IV	17 (44)	8 (73)		13 (46)	12 (55)		9 (37)	16 (62)			9 (53)	16 (55)
**Tumor size, n (%)**													
>4 cm	5 (13)	2 (18)	0.9686	4 (14)	3 (14)	0.7302	4 (17)	3 (12)	0.9091		4 (19)	3 (10)	0.6438
≤4 cm	34 (87)	9 (82)		24 (86)	19 (86)		20 (83)	23 (88)			17 (81)	26 (90)
**Lymph node, n (%)**													
Positive	14 (36)	5 (45)	0.8219	11 (39)	8 (36)	0.8327	8 (33)	11 (42)	0.5137		7 (33)	12 (41)	0.5629
Negative	25 (64)	6 (55)		17 (61)	14 (64)		16 (67)	15 (58)			14 (67)	17 (59)
**Nerve invasion, n (%)**													
Positive	32 (82)	9 (82)	0.6697	24 (86)	17 (77)	0.6888	21 (88)	20 (77)	0.5457		18 (86)	23 (79)	0.8346
Negative	7 (18)	2 (18)		4 (14)	5 (23)		3 (12)	6 (23)			3 (14)	6 (21)
**Vascular invasion, n (%)**													
Positive	13 (33)	8 (73)	0.0464*	12 (43)	9 (41)	0.8898	9 (38)	12 (46)	0.5357		9 (53)	12 (41)	0.9168
Negative	26 (67)	3 (27)		16 (57)	13 (59)		15 (62)	14 (54)			12 (57)	17 (59)

*Statistical significance.

Surgical resection was performed in all patients as the sole treatment for 20 patients (40.0%). Thirty (60.0%) received adjuvant chemotherapy after surgery. Eight (16.0%) patients had ≥2 lines of chemotherapy. At the time of last follow-up, 19 patients (38.0%) experienced local (two, 10.5%) or distant recurrences (17, 89.5%) postoperatively.

### Mutant Prevalence of Stromal Components

**Figure 1:**
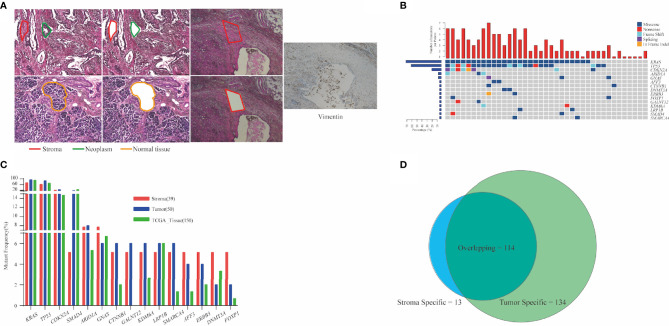
Analysis of mutation detection in stromal components. **(A)** Representative schematic diagram showing the three components of PDAC: the neoplastic, stroma, and normal pancreatic components. Vimentin was enriched in stromal components. **(B)** Mutational prevalence of stromal components. Top 15 genes mutated in over two stroma specimens are shown. In total, 127 somatic mutations were detected in stromal components from 39 patients. *KRAS* (71.8%), *TP53* (61.5%), and *CDKN2A* (23.1%) were the most recurrent mutant genes. Top bars indicate the number of mutations. Left-hand bars represent the frequency of each gene. Hot plot shows the detailed mutations detected in each patient. **(C)** Comparison of mutant frequencies of top 15 genes in stroma, tumor, and public TCGA database. Altered *KRAS* and *TP53* were the most commonly altered genes and demonstrated concordance in three cohorts. **(D)** Common mutations in stromal and matched neoplastic components; 89.8% mutations in stroma also co-existed in matched neoplastic components. Thirteen stromal mutations were absent in matched neoplastic components, while 134 mutations were exclusive to the neoplastic components. PDAC, pancreatic ductal adenocarcinoma; TCGA, The Cancer Genome Atlas.

Cellular morphology was determined *via* H&E staining, and the incisal margin of LCM was kept away from nests as much as possible to avoid the contamination of neoplastic cells. As a specific biomarker of stroma-derived cells, vimentin was enriched in stromal components isolated from the adjacent section (**Figure 1A**). We evaluated the mutant prevalence in the stromal components. In total, 127 somatic mutations (median = 3, ranged from 1 to 7) were detected in stromal components from 39 patients **(** **Table S1** **)**. *KRAS* (n = 28, 71.8%), *TP53* (n = 24, 61.5%), and *CDKN2A* (n = 9, 23.1%) were the most recurrent mutant genes (**Figure 1B**). All of *KRAS* mutations were located in the hot spot codons 12 and 61, including G12C (n = 1), G12D (n = 11), G12R (n = 2), G12V (n = 12), Q61H (n = 2), and Q61R (n = 1) (**Figure S1**). Two *KRAS* mutations (G12R and G12V) co-existed in P05. Twenty-one *TP53* mutations (87.5%) occurred in DNA-binding domain (**Figure S1**). As the top two prevalent mutant genes, *KRAS* and *TP53* were co-altered in 21 patients (**Figure 1B**).

Droplet digital PCR (ddPCR) was used to verify the credibility of stromal mutations, and the most common mutation site (*KRAS* G12D/V) from seven stromal specimens with different VAF range was selected. As the results, all of seven mutations were indeed repeated *via* ddPCR, and the VAFs estimated by ddPCR and NGS demonstrated strong consistency (R^2^ = 0.9067, *p* = 0.0009, **Figure S2**).

Correlation between clinical characteristics and genomic alterations of the stroma was then explored. The stroma was more likely to be genomically altered in patients without vascular invasion than in those with vascular invasion (*p* = 0.0464, **Table 1**). Mutants *KRAS* (*p* = 0.0036) and *TP53* (*p* = 0.0101), as well as co-mutants *KRAS* and *TP53* (*p* = 0.0029), more likely occurred in older patients than in younger ones, although the distribution of age at diagnosis suggests no discrepancy between patients with and without stromal mutations (**Table 1**). Nevertheless, there was no significant correlation between other baseline characteristics and mutations in stroma (**Table 1**).

### Mutational Overlap Between Stromal and Matched Neoplastic Components

Next, a total of 248 mutations were detected from 50 neoplastic specimens (median = 5, ranged from 1 to 11, **Table S1**). Commonly altered genes in neoplastic components included *KRAS* (n = 47, 94.0%), *TP53* (n = 43, 86.0%), *CDKN2A* (n = 12, 24.0%), and *SMAD4* (n = 9, 18.0%) (**Figure S3**). We validated the mutation prevalence of both stromal and neoplastic components using TCGA data. Altered *KRAS* and *TP53* were the most commonly altered genes and demonstrated concordance in three cohorts, with less prevalence in the stroma than in the other two cohorts (71.2% and 61.5% in stroma, 94.0% and 86.0% in tumor, and 90.7% and 69.3% in TCGA) (**Figure 1C**).

Stromal mutations were further verified in matched neoplastic components. Overall, 114 mutations co-existed in both components. Thirteen stromal mutations were absent in matched neoplastic components, while 134 mutations were exclusive to the neoplastic components (**Figures 1D**, **S3**). Critical driver gene analysis and function prediction showed 71 candidate driver events (62.3%, 71/114) that occurred in nine genes, including *KRAS*, *TP53*, *CDKN2A*, *ARID1A*, *GNAS*, *KDM6A*, *RNF43*, *SMAD4*, and *TGFBR2* (**Figure S4**). All mutations of *KRAS* and *TP53* in stroma were also identified in matched neoplasm, except one *KRAS* mutation in P05, which harbored two different *KRAS* mutations in the stroma (**Figure S3**). Furthermore, *KRAS* mutations identified in the neoplastic components were absent in matched stroma for 19 patients (38.0%). The same *TP53* mutations were identified in matched neoplastic components of only 19 patients (38.0%) (**Figure S3**). For 13 stroma-specific mutations, there were only two driver events (15.4%, *KRAS* G12V for P05, *SMAD4* W302* for P43, **Table S1**), while 57 driver events (42.5%) were underlined in 134 neoplasm-specific mutations. We further performed pathway enrichment analysis for stroma-specific and neoplasm-specific mutations using Kyoto Encyclopedia of Genes and Genomes (KEGG) resource. As a result, a more intimate connection with tumorigenesis and development was highlighted for neoplasm-specific mutations compared with stroma-specific mutations (**Figure S5**). Those co-existed mutations in both components indicated that cancer cells affected the genomic features of stroma *via* EMT. On the other hand, those non-tumor-related mutations in stromal components demonstrated that genomic variants indeed occurred in TME, which might be attributed to clonal evolution.

### Clonality Analysis of Mutations

**Figure 2:**
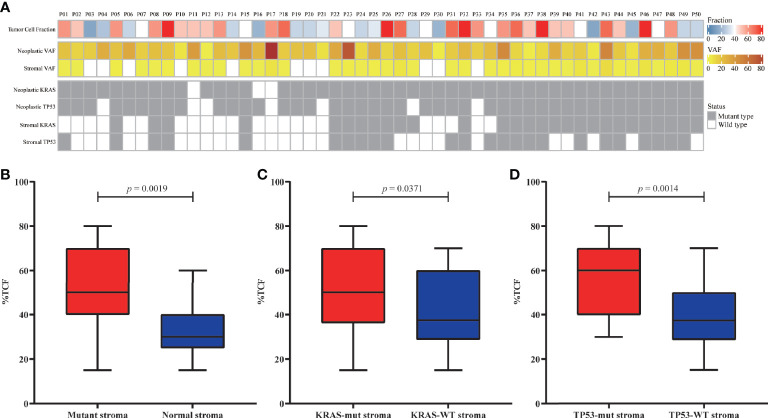
Correlation between the tumor cell fraction (TCF) and genomic status. **(A)** Overview of the TCF, stroma/neoplastic VAF, and *KRAS/TP53* status for each patient. **(B–D)** Comparison of the TCF from patients with mutant or normal stroma **(B)**, with or without *KRAS* mutation **(C)**, and with or without *TP53* mutation **(D)**. VAF, variant allele frequency.

The TCF could impact the analysis of clonality. The correlation between TCF estimated by microimaging and the mutational status of the neoplasm and stroma were evaluated (**Figure 2A**). The TCF in patients with mutant stroma was significantly higher than in those with normal stroma (*p* = 0.0019, **Figure 2B**). However, the VAFs in neither stroma (**Figure S6A**) nor neoplasm (**Figure S6B**) were correlated with the TCF. Patients with *KRAS/TP53*-mut stroma also demonstrated a higher TCF than did those with wild-type stroma (*p* = 0.0371, *p* = 0.0014, **Figures 2C, D**). Nevertheless, no discrepancy was seen for patients with or without *KRAS/TP53*-mut neoplasm (**Figure S7**), possibly due to the extremely high detection rate of *KRAS* and *TP53* mutation in neoplastic components.

**Figure 3:**
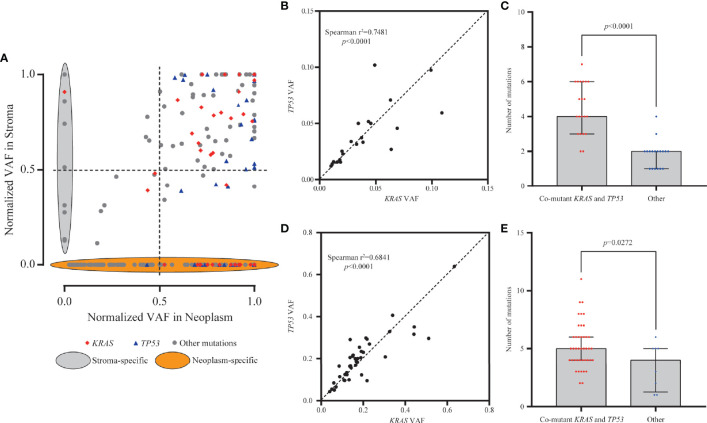
Clonality and mutation burden analysis of *KRAS* in stromal and neoplastic components. **(A)** Variant allele frequencies (VAFs) of each mutation were normalized with maximum VAF in the same specimen to assess the clonality. Mutations with ≥50% normalized VAFs were more likely to be clonal events; 86.8% of common mutations were clonal events in stroma. **(B, D)** The VAFs of *KRAS* and *TP53* mutations showed statistically significant consistency in the stromal (Spearman’s r^2^ = 0.7481, *p* < 0.0001) and neoplastic (Spearman’s r^2^ = 0.6841, *p* < 0.0001) components, indicating that mutants *KRAS* and *TP53* might have co-occurred around the same early period. **(C, E)** Samples with co-mutants *KRAS* and *TP53* tended to harbor more mutations than the other mutant subtype samples, in both stromal (*p* < 0.0001) and neoplastic (*p* = 0.0272) components.

To eliminate the frequency bias between neoplastic and stromal mutations, we normalized the VAFs of each mutation (absolute VAF/maximum VAF in the same specimen) in paired stromal and neoplastic components. Mutations with ≥50.0% normalized VAFs were more likely to be clonal events and occurred early during development of stroma or neoplasm than those with <50.0% normalized VAFs. For 114 mutations common in both components, 99 mutations (86.8%) were clonal events in stroma, among which 96 (84.2%) presented ≥50.0% normalized VAFs in both neoplasm and stroma (**Figure 3A**). However, for 134 neoplasm-specific mutations, only 72 (53.7%) were clonal in neoplastic components (**Figure 3A**), significantly lower than the proportion of common mutations (χ^2^ *p* < 0.0001).

In neoplastic and stromal components, 95.8% (46/48) and 89.7% (26/29) of *KRAS* mutations were clonal, respectively (**Figure 3A**). For mutant *TP53*, 97.7% (42/43) in neoplasm and 87.5% (21/24) in stroma were clonal variants (**Figure 3A**). The VAFs of *KRAS* and *TP53* mutations in the same stroma presented statistically significant consistency (Spearman’s r^2^ = 0.7481, *p* < 0.0001, **Figure 3B**), indicating that mutants *KRAS* and *TP53* might have co-occurred around the same early period. Moreover, stroma with co-occurred *KRAS* and *TP53* tended to harbor more mutations in stroma than the others (*p* < 0.0001) (**Figure 3C**). Similarly in neoplastic components, *KRAS* and *TP53* often co-altered and showed significant consistency (Spearman’s r^2^ = 0.6841, *p* < 0.0001, **Figure 3D**). More mutations were detected in neoplasm with co-mutations than those without co-mutations (*p* = 0.0272, **Figure 3E**). Altogether, these results indicated that the neoplasm and adjacent stroma were likely to share the similar clonal trunk events, especially for mutants *KRAS* and *TP53*, during the early tumorigenesis.

**Figure 4:**
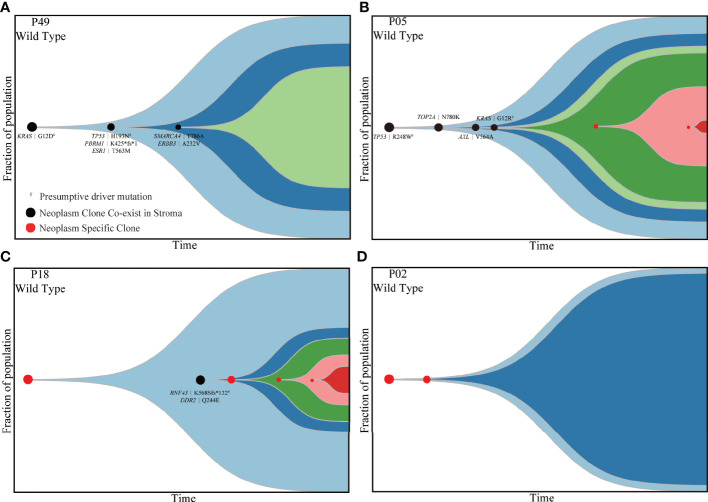
Four types of patients demonstrating different evolutionary trajectories involving both stroma and neoplastic components. P49 **(A)**, P05 **(B)**, P18 **(C)** and P02 **(D)** presented four different subtypes of evolutionary trajectory. The black dots indicate clones shared by matched stroma and neoplasm. The red dots represent clones private to neoplastic components. The black characters indicate mutations shared by matched stromal and neoplastic components. The emergence and progression pattern of each clone are hypothesized according to the fraction of clonal population inferred from the average VAF of mutations involved in the same clone. VAF, variant allele frequency.

Subsequently, PyClone strategy was utilized to reconstruct the evolutionary trajectory for neoplastic components. Ultimately, four subtypes were clarified for patients according to the genetic and driver imprinting derived from neoplasm upon stroma. The first type (A) included 12 patients characterized by the overall genomic and evolutional concordance, indicating that all neoplastic clones and driver events could be identified in stroma (**Figures 4A**, **S8A**). The second type (B) included 20 patients, and all stroma harbored the neoplastic initial clones and driver events but lacked some of subsequent clones (**Figures 4B**, **S8B**). The third type (C) involving five patients demonstrated total absence of neoplastic mutations in stroma (**Figures 4C**, **S8C**). The fourth type (D) included only two patients. For the first patient, the initial clone was absent, but a driver mutation (*RNF43* K568Sfs*132) involved in a latter clone was identified in stroma (**Figure 4D**). For the second patient, the initial neoplastic clone indeed expressed in stroma, but no driver mutation was identified (**Figure S8D**). Of note, patients with mutants *KRAS* and *TP53* in stroma were either type A or B. There was no significant discrepancy of TCF among the four different subtypes (**Figure S9**). Overall, type A/B was defined as neoplasm-like stroma based on the similar genetic performance and evolutional entanglements between neoplastic and stromal components.

### *KRAS* Mutation Promotes Mesenchymal Transformation of Epithelial Cancer Cell

**Figure 5:**
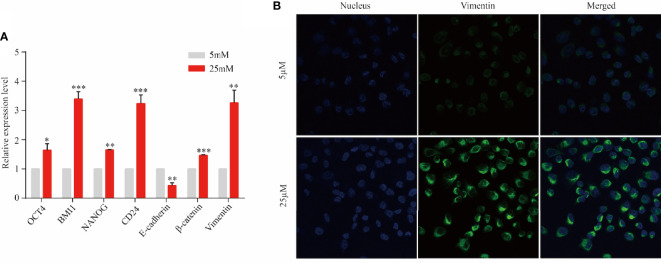
*KRAS*-mutant tumor cells might transform into stem-like cells. Panc1 cells were cultured for 3 days in the presence of 5 or 25 µM of dextrose. **(A)** The expression level of OCT4, BMI1, NANOG, SOX2, CD24, E-cadherin, β-catenin, and vimentin was detected by RT-PCR. Data are presented as mean ± SEM of three independent experiments. **(B)** Vimentin expression was analyzed by immunofluorescent staining (blue, nucleus; green, vimentin). Original magnification ×600. *p < 0.05, **p < 0.005, ***p < 0.0005.

Epithelial cancer cell were observed to migrate into the stroma and transformed to mesenchymal phenotype *via* EMT in a PanIN mouse model carried *KRAS* mutation (9). Based on this study and aforementioned findings, multi-lineage differentiation potential and mesenchymal transformation of *KRAS*-mutant cell line Panc1 were evaluated to validate this result. After 3 days of cell culture in DMEM with 5 and 25 µM of dextrose, five regulator markers of cell pluripotency involving OCT4, BMI1, NANOG, SOX2, and CD24 were detected by RT-PCR. These five markers exhibited significant increase in the 25 µM group compared with the 5 µM group (**Figure 5A**). Furthermore, the expression of E-cadherin was significantly decreased while β-catenin was significantly increased in a high-nutrient environment, indicating the progress of EMT and cell migration (**Figure 5A**). Subsequently, immunofluorescent staining revealed an enhanced expression of vimentin in the 25 µM dextrose group but absent in the 5 µM dextrose group (**Figure 5B**). Those findings indicated that a fraction of tumor cells harbor multi-lineage differentiation potential and that *KRAS* mutation might facilitate this process.

### Genomic Status of Stroma Associated With the Postoperative Survival of Pancreatic Ductal Adenocarcinoma

**Figure 6:**
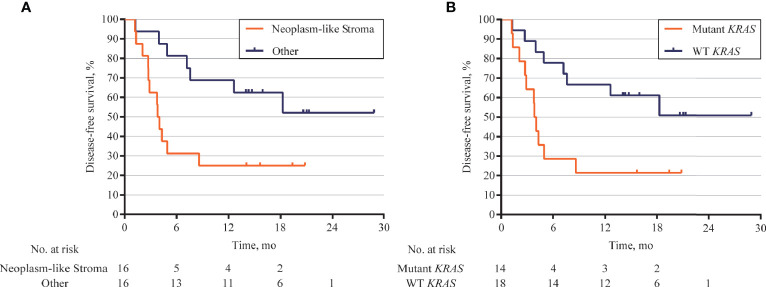
Kaplan–Meier curves for disease-free survival (DFS) according to genomic status of stroma. **(A)** DFS analysis in 32 patients followed up over 1 year after surgical operation. Patients with neoplasm-like stroma had markedly short DFS (median = 3.9 months) than those without neoplasm-like stroma (median DFS unreached, hazard ratio = 3.079, 95% CI 1.126 to 7.215, *p* = 0.0193). **(B)** Patients with *KRAS*-mutant stroma also showed poor DFS (median = 3.9 months) than those with *KRAS*-wild-type stroma (median DFS unreached, hazard ratio = 3.304, 95% CI 1.247 to 8.751, *p* = 0.0162).

The association between DFS and genomic status of stroma, as well as multiple clinicopathologic risk factors of PDAC, was analyzed for 32 patients who were followed up over 1 year after surgical operation. The Kaplan–Meier analysis showed that 32 patients with neoplasm-like stroma had a markedly reduced DFS time (median = 3.9 months) than had the other patients (median DFS unreached, hazard ratio = 3.079, 95% CI 1.126 to 7.215, *p* = 0.0193, **Figure 6A**). We further evaluated whether stromal *KRAS* mutations were associated with postoperative survival. As the results, patients with *KRAS*-mutant stroma had a significantly poorer DFS time (median = 3.9 months) than those with *KRAS*-wild-type stroma (median DFS unreached, hazard ratio = 3.304, 95% CI 1.247 to 8.751, *p* = 0.0162, **Figure 6B**), which was also confirmed by multivariate analysis (hazard ratio = 2.962, 95% CI 1.174 to 7.471, *p* = 0.021, **Table 2**). Patients with *TP53*-mutant stroma also showed poorer DFS time (median = 3.8 months) than those with *TP53*-wild-type stroma (median DFS unreached, hazard ratio = 3.143, 95% CI 1.112 to 8.880, *p* = 0.0307). However, although exhibiting a certain trend in univariate analysis, all clinicopathologic risk factors of PDAC, including clinical stage, tumor size, histological differentiation, lymph node involvement, nerve, and vasculature invasion, showed no significant association with DFS in univariate and multivariate analyses (**Figure S10**, **Table 2**).

**Table 2:** Univariate and multivariate Cox analyses for risk factors of relapse.

Variables	Univariate analysis		Multivariate analysis	
	HR (95% CI)	*p*	HR (95% CI)	*p*
Clinical stage, II/IV *vs*. I	0.73 (0.30–1.80)	0.492	–	–
Tumor size, >4 *vs*. ≤4 cm	0.53 (0.13–2.16)	0.378	–	–
Differentiation, poor *vs*. other	2.00 (0.80–4.87)	0.142	–	–
Lymph node metastasis, Positive *vs*. negative	2.05 (0.75–5.62)	0.164	–	–
Nerve invasion, positive *vs*. negative	2.43 (0.89–6.65)	0.085	–	–
Vascular invasion, positive *vs*. negative	1.93 (0.76–4.92)	0.169	–	–
Adjuvant chemotherapy, positive *vs*. negative	0.90 (0.36–2.28)	0.831	–	–
*KRAS* status in stroma, Mutant type *vs*. wild type	3.30 (1.25–8.75)	0.016*	2.96 (1.17–7.47)	0.021*,^a^

*Statistical significance.

a Multivariate analysis was performed using method Forward: LR.

## Discussion

The tumor stroma has important roles in cancer development, progression, and metastasis (17). Although previous studies demonstrated the complex biophysical and transcriptional properties of stroma for patients with PDAC (18, 19), little evidence supports the clinical relevance of stroma genomic characteristics at present. In this study, we hypothesized that genomic mutations existed in stroma and might contribute to the clinical outcome of resectable PDAC. We identified 127 somatic mutations in stromal components separated by LCM methods and found *KRAS* mutations were highly prevalent and widely clonal in stroma. Subtyping based on genomic features, neoplasm-like, and *KRAS*-mutant stroma was associated with poor DFS.

As its first objective, our study initially reported the genomic alterations in the stroma and defined four subtypes according to the genetic and driver imprinting derived from neoplasm upon stroma for patients with PDAC. In view of the substantial mutations shared by tumor and stroma and the clonal relationship of two components, the stromal cells with shared mutations derived from the common progenitor with neoplastic cells, supporting that tumor cells affected the genomic features of stroma *via* EMT. Interestingly, stromal and neoplastic cells also experienced divergent evolution because of the emerged private genetic variants in both components. Rhim et al. reported that tagged epithelial cells invaded stroma prior to tumor formation and transformed to mesenchymal phenotype at early stage of PDAC, which is consistent with our results (9). Although the cellular morphology persists as “normal stromal cells,” the genetically and functionally neoplasm-like variants indicate the real status of stroma and distinguish different subtypes of PDAC for prognostic prediction. Activated stroma could promote the acquisition of more genetic and epigenetic changes in tumor cells and induce cancer development (20, 21). On the other side, autonomously genomic changes in the stroma were also suggested to induce tumorigenesis (22).

As a critical driver gene in early tumorigenesis (23), *KRAS* mutations were detected in stromal components from 28 PDAC (71.8%) cases, and 89.7% of these mutations were clonal events. Rhim et al. also reported that epithelial cancer cells can transform to mesenchymal phenotype and invade stroma in a PanIN mouse model that carried *KRAS* mutation (9). Based on these results, *KRAS*-mutant tumor cells might have more aggressive behavior on tumor progression and then affect the stroma genomic features *via* EMT; thus, we conducted some experiments to validate this condition *in vitro*. Results showed that *KRAS*-mutant tumor cells harbored higher multi-lineage differentiation potential and promote tumorigenesis *via* EMT, which was consistent with the above hypothesis. However, further mechanism needs to be explored by future fundamental researches, which can help in clarifying the underlying mechanisms and thus improving the therapeutic strategies for PDAC patients.

The outcome for resectable PDAC remains dismal despite improvements in surgical and oncological management strategies. In many cases, patients with similar clinicopathologic characteristics benefit variably in surgery outcome. Also, there is a lack of clear clinicopathologic evidence to guide clinicians to determine the therapeutic options before and after resection. Nowadays, resectability of PDAC has traditionally been assessed with geometric descriptions of the tumor–vessel interface (24). Despite recent therapeutic improvements, postoperative prognosis of PDAC remains very poor (25). Many clinicopathologic and serologic markers have been tested, but none is highly prognostic for PDAC patients (26). The genetic involvement between tumor and stroma seems associated with PDAC prognosis. In this study, we found that patients with *KRAS*-mutant stroma had a considerable risk of postoperative recurrence, and their survival was evidently worse than that of the other patients. If our results can be corroborated in a much larger prospective study, then analysis of driver mutation in PDAC stroma might help to guide a more precise treatment paradigm in adjuvant therapeutic options. For patients with neoplasm-like stroma, cutting off the crosstalk between neoplasm and stroma before conventional agents might improve their poor performance with PDAC. Furthermore, 94% patients harbor *KRAS* mutation in neoplasm, and prognosis of PDAC due to the extremely high mutant frequency failed to be predicted. Therefore, this stroma biomarker had evident advantage over conventional and tumor biomarkers, such as a more precise and accurate distinction of tumor prognosis.

Considering the complex immune environment in the stroma, another possible reason for worse prognosis is that such cancer-associated driver mutations in stroma may act to attenuate immune responses (27). Actually, it has been reported that mutations in the *KRAS* will activate the MAP kinase pathway and thus decrease the transcription of major histocompatibility complex class I molecules as well as the expression of other genes encoding molecules that are essential for peptide loading (28). In this case, cells harboring driver mutations might escape from immune response and act as the “bridgehead” to invade adjacent tissue and metastasize. These alterations might reduce inflammation in tumors and the killing of tumor cells by decreasing the density of T-cell ligands on tumor cells. The stiff extracellular matrix in stroma has a role as the bridge mediating the interactions between neoplasm and stroma, as well as the protector of tumor cells away from immune clearance and pharmacological effects (29). Therefore, several pro-fibrotic growth factors, such as TGF-β, PDGF, EGFR, and IGF-1, can be recruited as potential therapeutic targets to abolish such interactions and protections. Followed by these novel efforts, conventional chemoradiotherapy might achieve a much better effect for PDAC patients. Based on this finding, it may be useful to investigate the immune status of stroma for better understanding the prognostic effect of stromal *KRAS* mutations, which is in our plan.

Some limitations of this study persist. First, the follow-up time was short for some patients, and it still did not reach the median of overall survival, so the data were not efficiently utilized yet. Second, the limited morbidity of PDAC and the attribute of single-center study resulted in the relatively small sample size. To solve these problems, we have planned a multi-center study based on available results. Overall DFS and postoperative DFS are the primary and second endpoints, respectively. In order to further verify the results, we drew in this study. Third, to establish clinical utility of detecting mutations in stroma, the techniques applied here would also require improvement to satisfy feasibility for routine use. However, despite these limitations, this study can clearly indicate the genetic interaction between neoplastic and stromal components due to the normalized experimental and analytical procedures.

In this study, we performed parallel genotyping of stromal and neoplastic components and evaluated the prognostic ability of stromal markers for PDAC patients. We clarified the hereditary and evolutional connection between neoplasm and stroma, explored a novel prognostic marker based on stromal genomic status, and validated that *KRAS*-mutant stroma cells might derive from tumor cells with multi-lineage differentiation potential and promote tumorigenesis *via* EMT. Although it is currently complex and beyond routine clinical use to obtain stroma by LCM technique, we hope these efforts will be helpful to improve clinical management for PDAC patients in the near future.

In conclusion, a considerable proportion of PDAC stroma exhibit cancer-associated driver mutations, and four molecular subtypes were clarified according to the evolutional connection between neoplasm and stroma. Stromal *KRAS* mutations may serve as prognostic biomarkers in resectable PDAC and might help to guide a more precise treatment paradigm in therapeutic options.

## Data Availability Statement

The datasets presented in this study can be found in online repositories. The names of the repository/repositories and accession number(s) can be found in https://www.ncbi.nlm.nih.gov/, PRJNA561217.

## Ethics Statement

The studies involving human participants were reviewed and approved by Zhejiang Provincial People’s Hospital (No. 2016KY129). The patients/participants provided their written informed consent to participate in this study.

## Author Contributions

JJ, YX, and LiuY contributed to conception and design of the study. JJ, XX and XY organized the database. JJ, GR, and LC performed the statistical analysis. JJ, YX, and LiuY wrote the first draft of the manuscript. JJ, YX, ZC, D-SH and LinY wrote sections of the manuscript. All authors contributed to manuscript revision and read and approved the submitted version.

## Funding

This work was supported by the National Natural Science Foundation of China (Nos. 81772575, 81972455) and the Foundation of Science Technology Department of Zhejiang Province (No. 2017C33116).

## Conflict of Interest

Authors YX, LC, XX, LinY, XY were employed by company Geneplus-Beijing Institute.

The remaining authors declare that the research was conducted in the absence of any commercial or financial relationships that could be construed as a potential conflict of interest.

## Publisher’s Note

All claims expressed in this article are solely those of the authors and do not necessarily represent those of their affiliated organizations, or those of the publisher, the editors and the reviewers. Any product that may be evaluated in this article, or claim that may be made by its manufacturer, is not guaranteed or endorsed by the publisher.
