# Supplementary material for: Genomic Landscape in Neoplasm-Like Stroma Reveals Distinct Prognostic Subtypes of Pancreatic Ductal Adenocarcinoma
Source: Front Oncol. 2021 Oct 18;11:771247. doi: 10.3389/fonc.2021.771247 (PMC8558555; doi:10.3389/fonc.2021.771247)
Supplement: Supplementary file 1 [file DataSheet_1.pdf]

Supplementary Figures

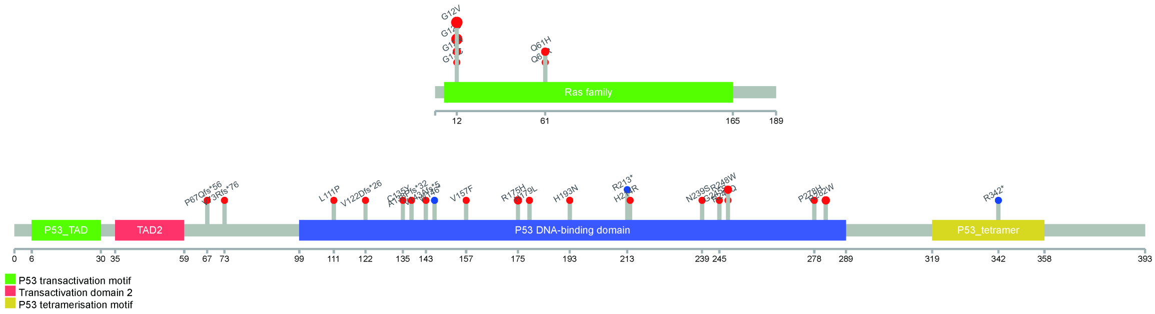

Fig. S1. Overview of mutant *KRAS* and *TP53* in stroma. The circle size indicates the number of mutations.

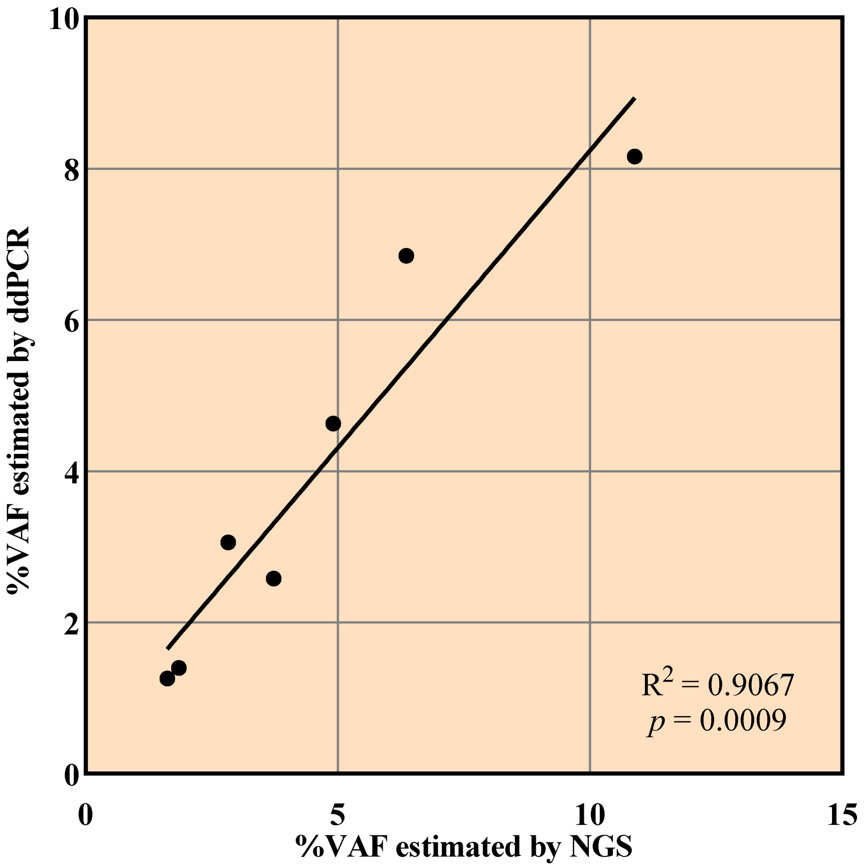

Fig. S2. Comparison of VAFs in stroma estimated by ddPCR and NGS. The correlation is calculated via Spearman algorithm. VAF, variant allele frequency; ddPCR, droplet digital PCR; NGS, next generation sequencing.

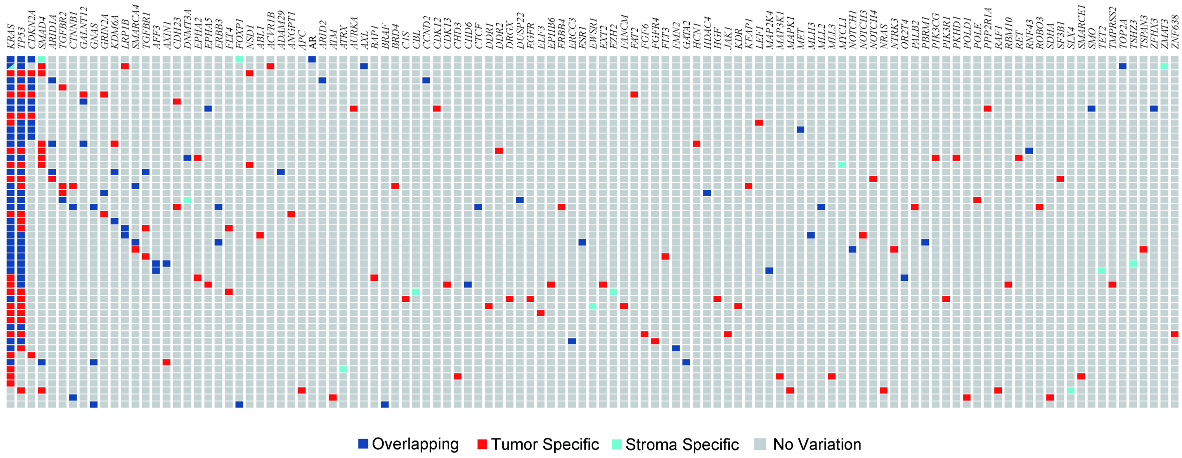

Fig. S3. Heatmap illustrating the mutational spectrum of each patient. Mutations are grouped according to their expression in matched tumor and stroma components.

a

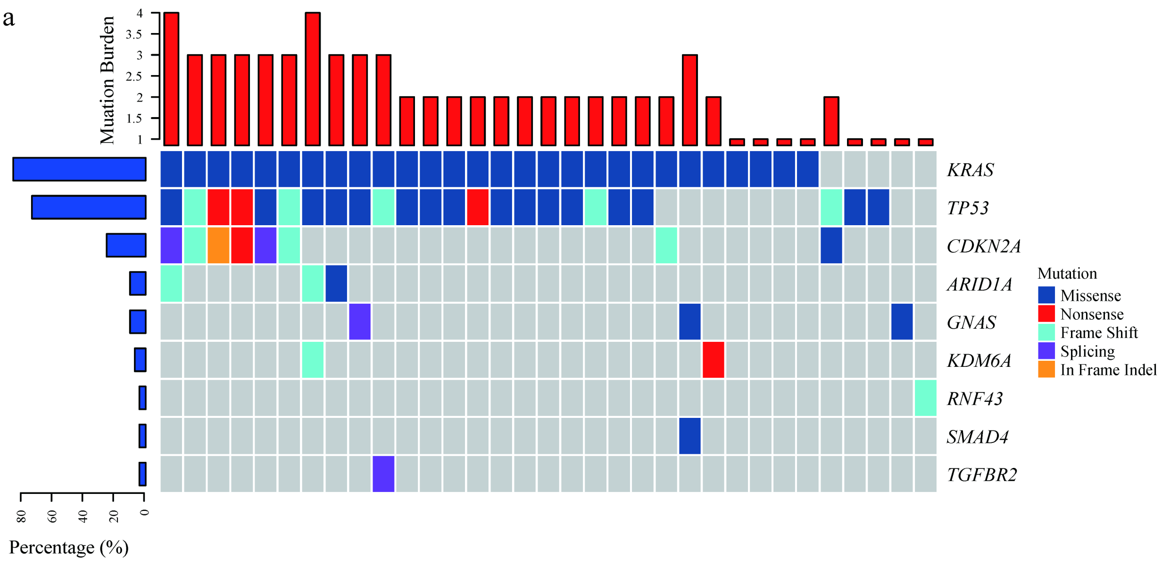

b

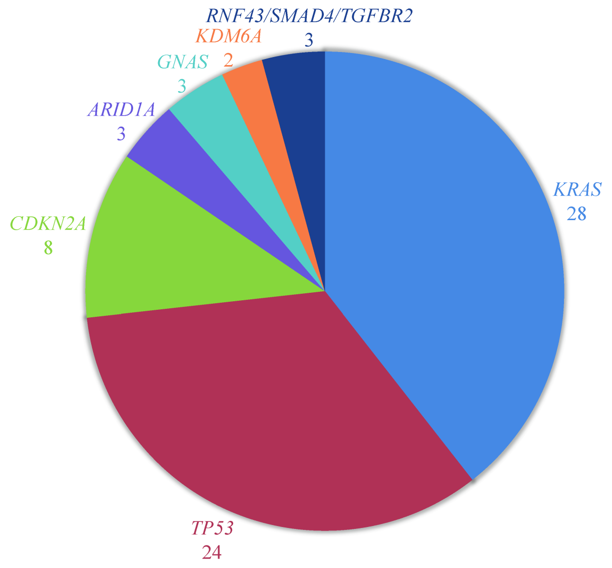

Fig. S4. The distribution of presumptive driver events in overlapping mutations between matched neoplastic and stromal components.

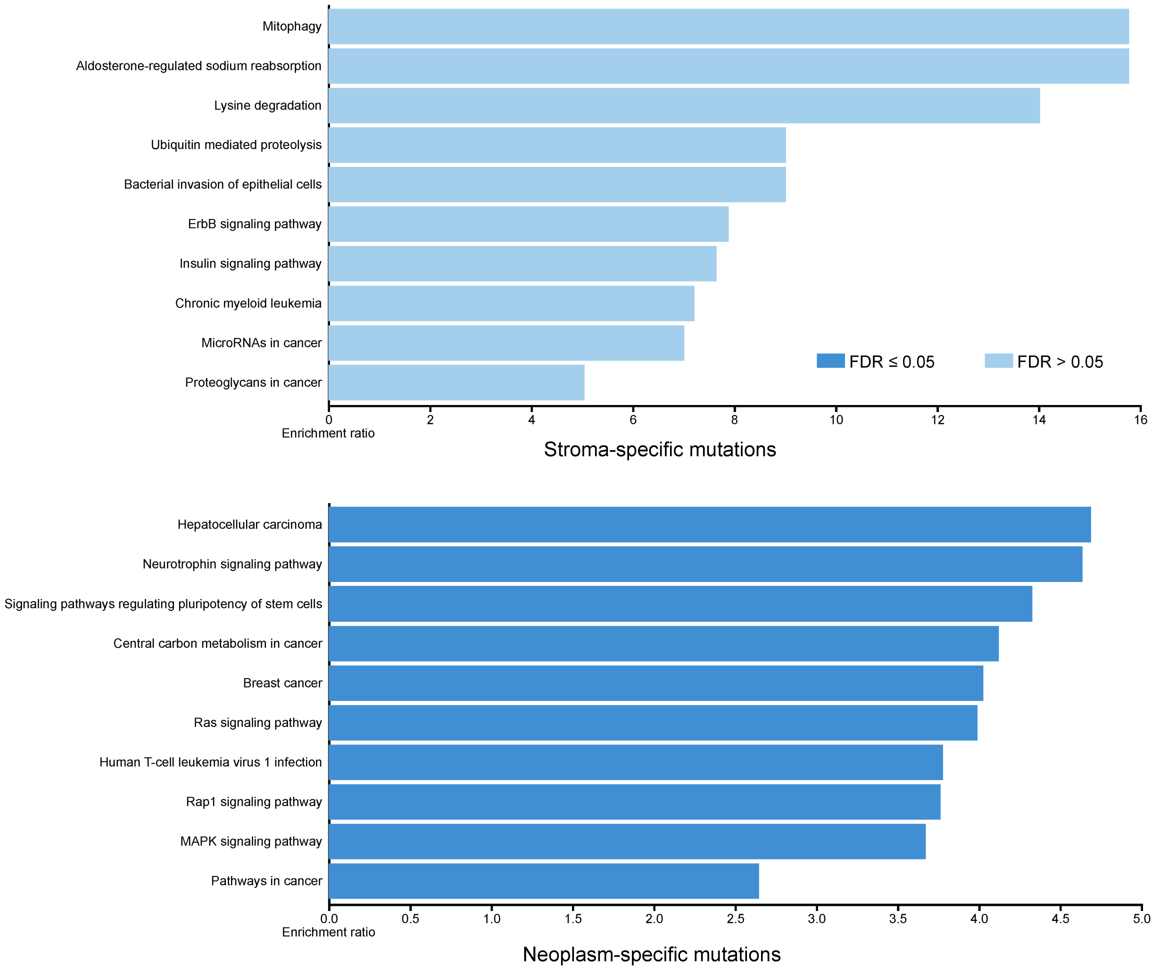

Fig. S5. Pathway enrichment analysis for stroma- and neoplasm-specific mutations. Kyoto Encyclopedia of Genes and Genomes resources were used. The plot was generated via web tool WebGestalt (http://www.webgestalt.org/option.php).

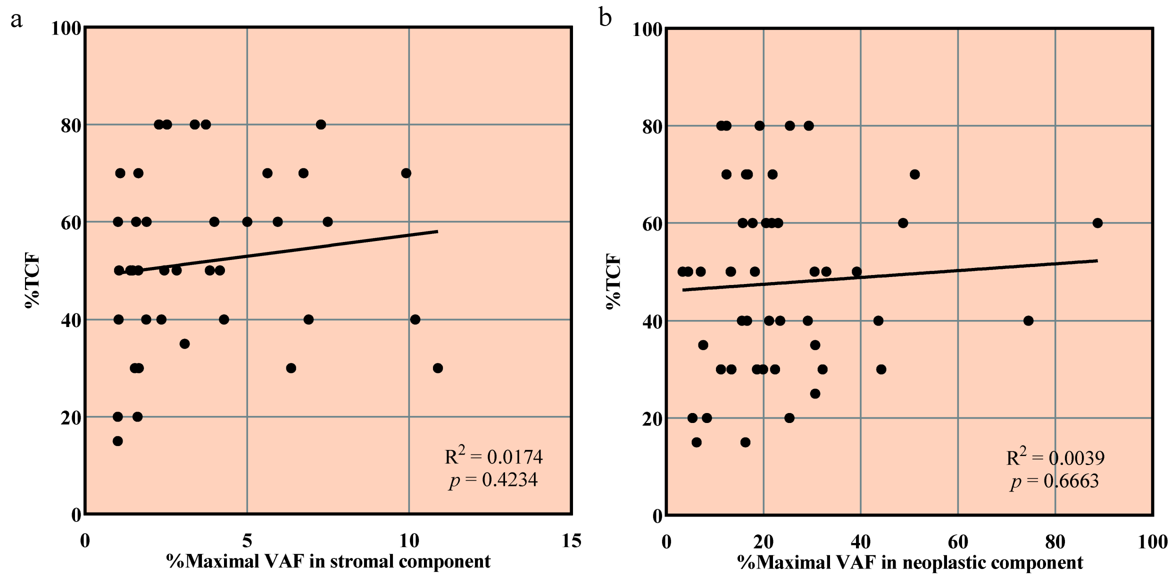

Fig. S6. Correlation between TCF and maximal VAF in stromal (a) or neoplastic component (b). The correlation is calculated via Spearman algorithm. Only components with mutations are included in this analysis. TCF, tumor cell fraction; VAF, variant allele frequency.

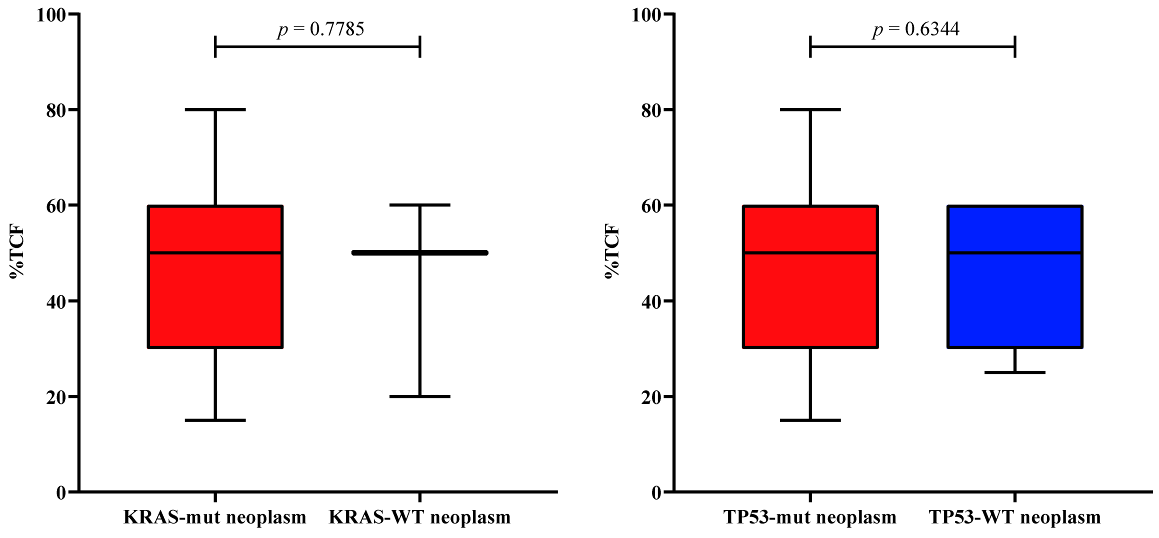

Fig. S7. Comparison of TCF between patients with KRAS/TP53-mut and WT neoplasm.

The group comparison is performed using Mann-Whitney U-test. TCF, tumor cell fraction.

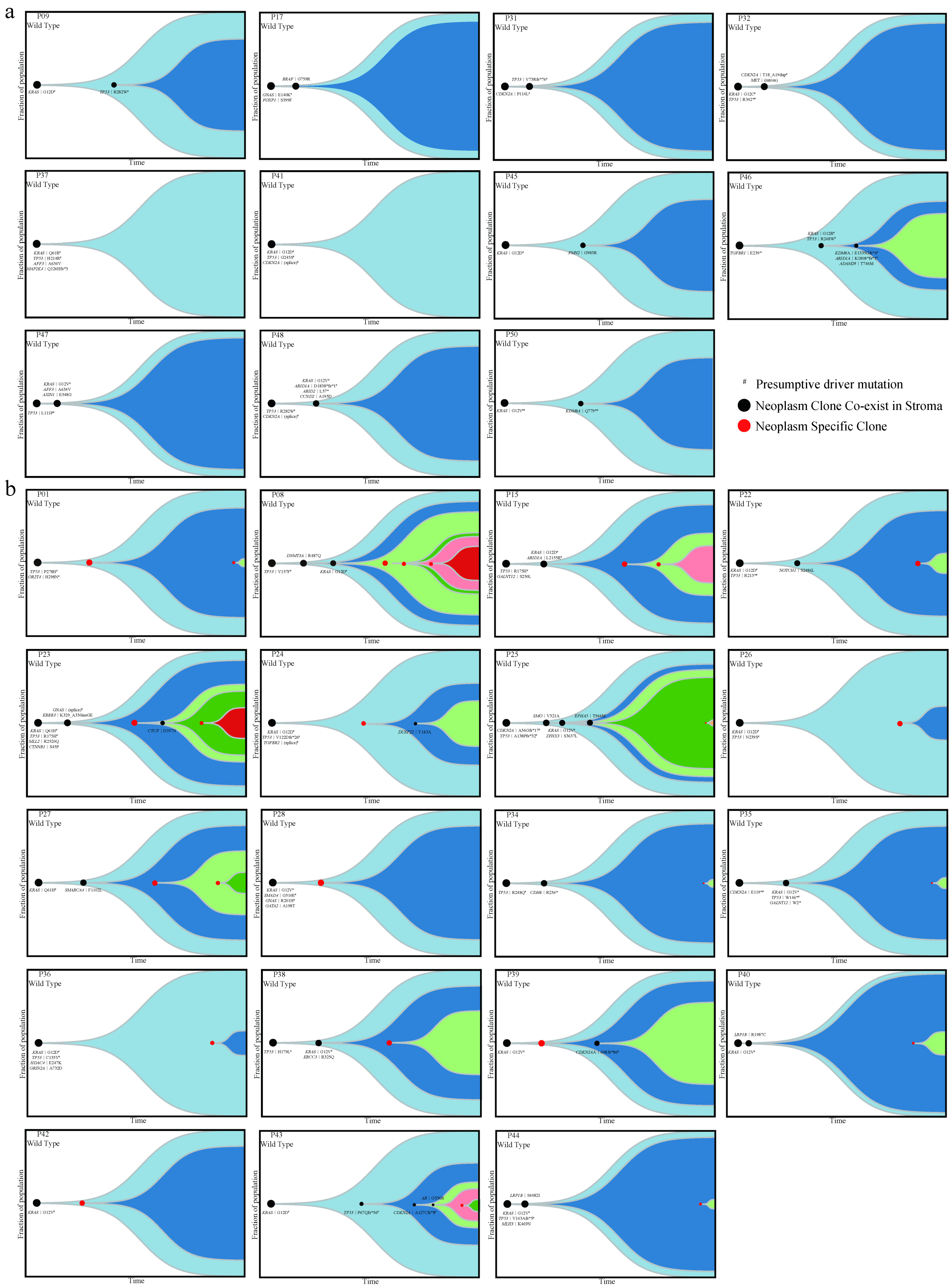
a
b
P09
P17
P31
P32
P37
P41
P45
P46
P47
P48
P50
P01
P08
P15
P22
P23
P24
P25
P26
P27
P28
P34
P35
P36
P38
P39
P40
P42
P43
P44
Wild Type
Fraction of population
Time
Presumptive driver mutation
Neoplasm Clone Co-exist in Stroma
Neoplasm Specific Clone

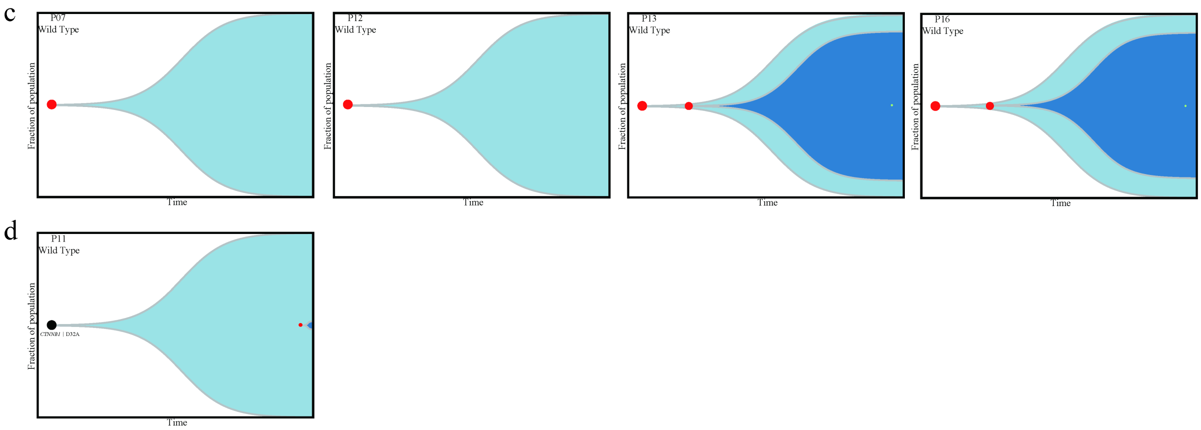

Fig. S8. Four types of patients demonstrating different evolutionary trajectories involving both stromal and neoplastic components. The black dots indicate clones shared by matched stroma and tumor. The red dots represent clones private to stromal components. The black characters indicate mutant genes in stromal components.

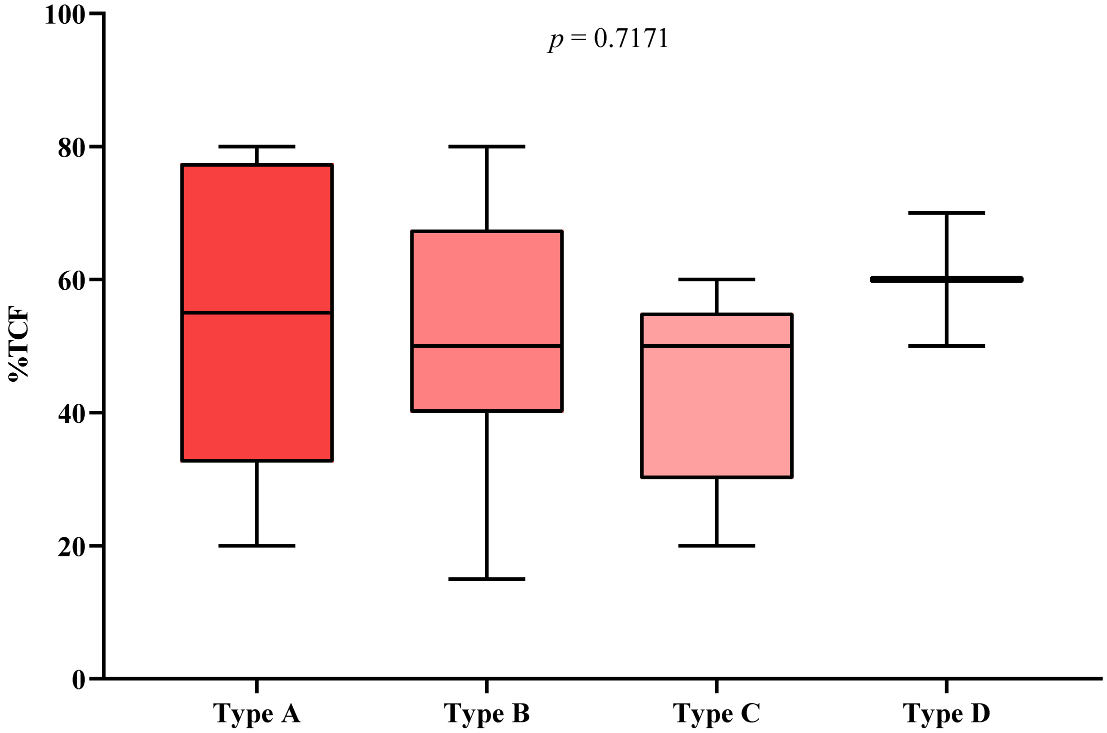

Fig. S9. Comparison of TCF among patients with different subtype stroma. The group comparison is performed using One-Way ANOVA test. TCF, tumor cell fraction.

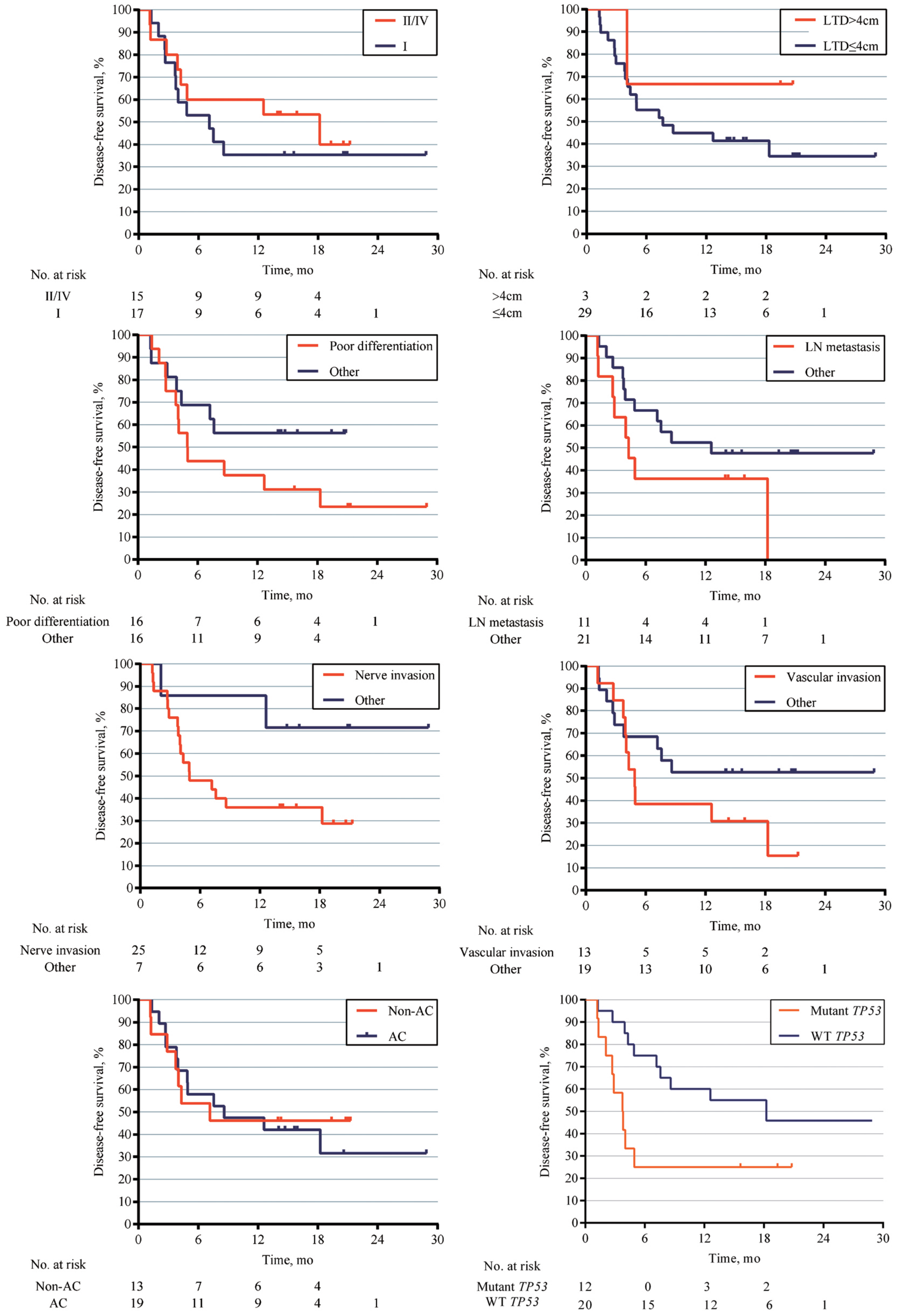

Fig. S10. Kaplan-Meier analysis between clinical risk factors and postoperative survival.

LTD, largest tumor diameter; LN, lymph node; AC, adjuvant chemotherapy.
